# A biosensing system using a multiparameter nonlinear dynamic analysis of cardiomyocyte beating for drug-induced arrhythmia recognition

**DOI:** 10.1038/s41378-022-00383-1

**Published:** 2022-05-09

**Authors:** Hao Wang, Yue Wu, Quchao Zou, Wenjian Yang, Zhongyuan Xu, Hao Dong, Zhijing Zhu, Depeng Wang, Tianxing Wang, Ning Hu, Diming Zhang

**Affiliations:** 1 State Key Laboratory of Optoelectronic Materials and Technologies, Guangdong Province Key Laboratory of Display Material and Technology, School of Electronics and Information Technology, Sun Yat-sen University, Guangzhou, 510006 China; 2 Research Center for Intelligent Sensing Systems, Zhejiang Lab, Hangzhou, 311121 China; 3 ZJU-Hangzhou Global Scientific and Technological Innovation Center, Department of Chemistry, The Second Affiliated Hospital Zhejiang University School of Medicine, Department of Clinical Medical Engineering, Zhejiang University, Hangzhou, 310058 China; 4 Key Laboratory of Novel Target and Drug Study for Neural Repair of Zhejiang Province, School of Medicine, School of Computer & Computing Science, Zhejiang University City College, Hangzhou, 310015 China; 5 School of Brain Science and Brain Medicine, Zhejiang University, Hangzhou, 310058 China; 6 College of Energy and Power Engineering, Nanjing University of Aeronautics and Astronautics, Nanjing, 210016 China; 7 E-LinkCare Meditech Co., Ltd., Hangzhou, 310011 China; 8 State Key Laboratory of Transducer Technology, Chinese Academy of Sciences, Shanghai, 200050 China

**Keywords:** Electrical and electronic engineering, Micro-optics

## Abstract

Cardiovascular disease is the number one cause of death in humans. Therefore, cardiotoxicity is one of the most important adverse effects assessed by arrhythmia recognition in drug development. Recently, cell-based techniques developed for arrhythmia recognition primarily employ linear methods such as time-domain analysis that detect and compare individual waveforms and thus fall short in some applications that require automated and efficient arrhythmia recognition from large datasets. We carried out the first report to develop a biosensing system that integrated impedance measurement and multiparameter nonlinear dynamic algorithm (MNDA) analysis for drug-induced arrhythmia recognition and classification. The biosensing system cultured cardiomyocytes as physiologically relevant models, used interdigitated electrodes to detect the mechanical beating of the cardiomyocytes, and employed MNDA analysis to recognize drug-induced arrhythmia from the cardiomyocyte beating recording. The best performing MNDA parameter, approximate entropy, enabled the system to recognize the appearance of sertindole- and norepinephrine-induced arrhythmia in the recording. The MNDA reconstruction in phase space enabled the system to classify the different arrhythmias and quantify the severity of arrhythmia. This new biosensing system utilizing MNDA provides a promising and alternative method for drug-induced arrhythmia recognition and classification in cardiological and pharmaceutical applications.

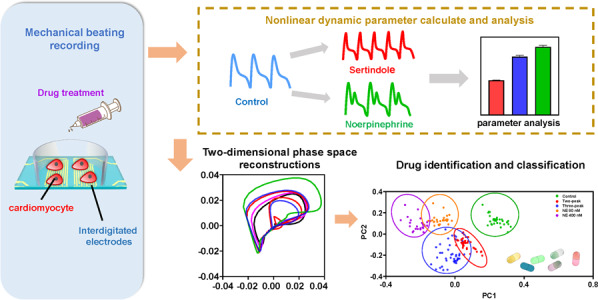

## Introduction

Cardiac arrhythmias consist of too fast, too slow, or irregular heartbeats, which are termed tachycardia, tachycardia and irregular pulsation, respectively^1–3^. Serious arrhythmias can lead to less effectiveness in heart pumps and may result in loss of heart function in sudden cardiac death (SCD), which can cause 17.9 million deaths worldwide every year^4,5^. Drugs treating noncardiac diseases can also cause cardiac arrhythmias in a number of clinical situations^6–8^. Considering the high mortality of SCD, cardiac arrhythmias caused by drugs have become one of the most crucial adverse effects to be assessed in novel drug development and postmarketing drug surveillance^9–11^. Despite the adverse effect assessments, up to now, ~45% of all drug withdrawals from the market are due to possible cardiotoxicity that may cause arrhythmias^12,13^. Hence, it is important to develop techniques to detect and analyze drug-induced arrhythmias in drug development to prevent drug-induced cardiac damage to human beings and avoid economic losses from drug recalls.

To assess drug-induced arrhythmias, researchers have developed several in vivo and in vitro techniques to record cardiac electrophysiology, such as self-powered ultrasensitive pulse sensors^14^ and bioresorbable triboelectric sensors^15^. Several in vivo techniques use animal models such as dogs, monkeys, and mice to obtain electrocardiograms and mirror drug-induced arrhythmias in clinical trials^16,17^. Although these techniques could closely report correlations between the drugs and arrhythmias, they are severely limited by the low economic and time efficiency of animal experiments required during large drug library screening. Moreover, step-by-step animal operations, such as drug injection and 12-lead electrocardiography, further increase the difficulty of achieving high-throughput arrhythmia detection. To overcome these limitations, cell-based electrophysiological techniques such as patch clamps, microelectrode arrays (MEAs), and interdigitated electrodes (IDEs) have emerged to culture cardiomyocytes in vitro as physiologically relevant models for arrhythmia detection^18–20^. Cell-based techniques take advantage of the electrophysiological properties of cardiomyocytes to capture drug-induced arrhythmias, thereby minimizing animal sacrifice and enabling low-cost electrophysiological recording in screening the arrhythmia risk for large drug libraries. Typically, cell-based techniques such as MEA and IDE can achieve simultaneous multichannel recording of membrane potential or mechanical beating signals, which further facilitates low-cost and high-throughput cardiac electrophysiological recording for drug-induced arrhythmia detection. Cell-based techniques have already been applied to investigate drug-induced arrhythmia and approved as a sensitive, robust, and efficient platform for testing drug effectiveness and for arrhythmia screening^21,22^.

The high efficiency of cell-based techniques such as the MEA and IDE have endowed them with a great capacity to predict arrhythmia risk by producing a large amount of data about membrane potential and mechanical beating^23–25^. However, most data analysis methods usually depend on conventional parameter extractions or naked eye examinations, which are less efficient in analyzing large datasets from MEA and IDE recordings^24,26^. Several studies have reported on automated template matching to improve the efficiency of arrhythmia recognition^27,28^. Although matching comparisons can report the existence of arrhythmias, they cannot provide the classification of arrhythmias. Thus, the development of data analyzing methods using cell-based techniques (e.g., MEA and IDE) lags behind the development of cell-based technique capacities that measure the data concerning membrane potential and mechanical beating for arrhythmia recognition. All these facts show that an analysis method for the data recorded by cell-based techniques is the next key point to fabricate more efficient biosensing systems for drug-induced arrhythmia assessment.

Nonlinear dynamics analysis is a promising method for information processing in numerous fields, including physics, engineering, biology, and medicine^29–31^. Most nonlinear approaches are based on the geometrical and topological analysis of the trajectories and attractors in phase space. Some commonly used nonlinear dynamics signal analysis methods include reconstructed phase space analysis, Lyapunov exponents, correlation dimension, approximate entropy, and sample entropy^32,33^. Recently, several new approaches via nonlinear dynamics analysis have been reported to process electrocardiogram (ECG) signals that present similar electrophysiological profiles with cell-based cardiac electrophysiological recording^34–36^. For example, nonlinear dynamics analysis has been employed to classify five different arrhythmias based on ECG data^37^. The nonlinear dynamics approaches can complement the traditional analysis that uses time- and frequency-domain linear methods by monitoring the dynamic change of ECG signals without individual waveform comparisons. However, to the best of our knowledge, no previous study has demonstrated applications of nonlinear dynamic analysis in cell-based cardiac electrophysiological recording or further drug-induced arrhythmia assessment of cell-based recording. It remains unanswered whether nonlinear dynamics analysis can work well on cell-based cardiac electrophysiological recording, especially on cardiac mechanical beating, whose profile differs from conventional cardiac electrophysiological signals.

**Fig. 1:**
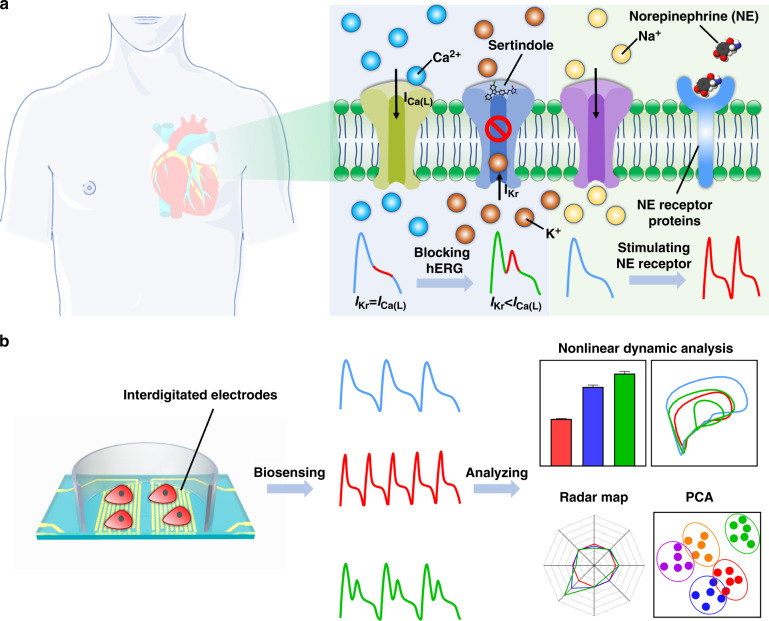
Schematic of the biosensing system integrating IDE impedance measurement and MNDA analysis for drug-induced arrhythmia recognition. **a** The drug-induced arrhythmia of cardiomyocytes. Sertindole blocks K^+^ channels on the cell membrane of cardiomyocytes, and norepinephrine (NE) increases the beating rate of cardiomyocytes. They both change the electrophysiological profiles of cardiomyocytes. **b** The biosensing system recognized arrhythmia from cardiomyocyte mechanical beating signals by MNDA analysis. The interdigitated electrodes collected the cardiomyocyte mechanical beating signals by the impedance measurement technique. The cardiomyocyte beating signals showed significant arrhythmias with the treatment of drugs including sertindole and norepinephrine. The MNDA analysis and PCA clustering recognized and distinguished the arrhythmias from the cardiomyocyte beating signals

Here, we developed a biosensing system that integrated the IDE impedance measurement and the multiparameter nonlinear dynamic algorithm (MNDA) analysis for drug-induced arrhythmia recognition and classification (Fig. 1). The IDEs were applied to detect the mechanical beating of human-induced pluripotent stem cell-derived cardiomyocytes (iPSC-CMs). Sertindole- and norepinephrine-induced arrhythmias were investigated due to their ability to cause *torsades de pointes* arrhythmia and sustained increases in human iPSC-CM beating frequency, respectively^21,38,39^. To explore whether MNDA analysis works for arrhythmia recognition, our work examined several nonlinear dynamics methods to generate ten MNDA parameters, including delay time, correlation dimension, embedding dimension, box dimension, largest Lyapunov exponent (LLE), Kolmogorov entropy, comentropy, approximate entropy, spectral entropy, and CO complexity, as candidates to analyze mechanical beating signals and recognize drug-induced arrhythmia. An MNDA parameter among the candidates, approximate entropy, showed a highly sensitive response to the occurrence of the arrhythmia induced by drug treatment. It was demonstrated that the approximate entropy can work well as a main indicator of MNDA analysis for both sertindole- and norepinephrine (NE)-induced arrhythmia, although these two drugs induced arrhythmia with different distorted profiles. To classify the different drug-induced arrhythmias, we reconstructed two-dimensional phase space to gain different plots for different drug-induced arrhythmias and further analyzed the plot with principal component analysis (PCA), which has enabled MNDA analysis to distinguish arrhythmias and quantify the severity of variant arrhythmias. To our knowledge, this is the first report to analyze cardiomyocyte mechanical beating signals with nonlinear techniques. This new biosensing system, by integrating the IDE impedance measurement and the MNDA analysis, can provide a promising and alternative method for the recognition and classification of drug-induced arrhythmia in cardiological and pharmaceutical applications.

## Experiments and methods

### IDE device fabrication

**Fig. 2:**
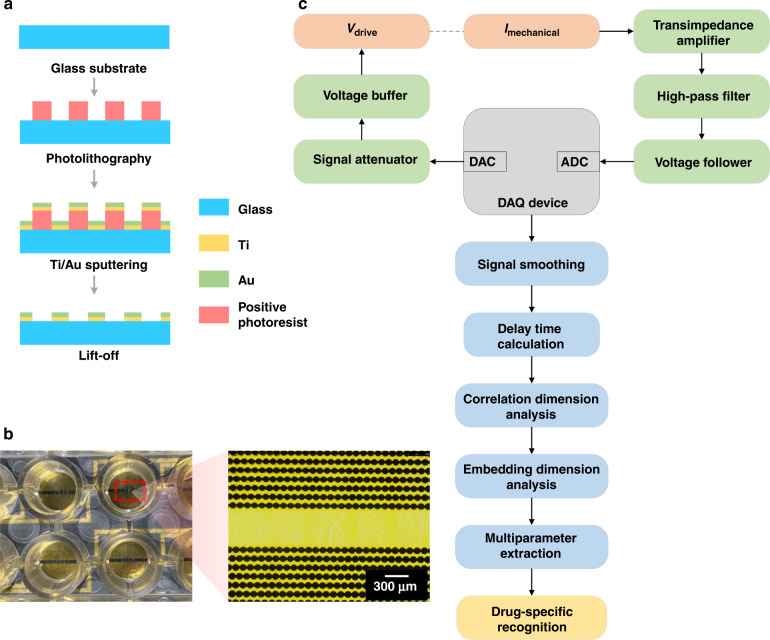
Design and implementation of the biosensing system combining IDE impedance measurements and MNDA analysis for drug-induced arrhythmia recognition. **a** The fabrication procedures of IDEs for cardiomyocyte mechanical beating recording by the impedance measurement technique. **b** Photo of the IDE devices and optical imaging of IDEs with a microscope. The red box shows the region of optical imaging of IDEs. **c** Block diagram of the biosensing system for arrhythmia recognition. The DAC module generated specific frequency sinusoidal voltages for the stimulation, and the ADC module received feedback signals for the calculation of the cardiomyocyte beating signals. Several algorithms, including MNDA analysis and PCA clustering, further worked on cardiomyocyte beating signals to achieve arrhythmia recognition and classification

The IDE device was fabricated by conventional microfabrication techniques, including photolithography, deposition, and liftoff processes (Fig. 2a). A 2 × 8 IDE array was fabricated on an 80 mm × 15 mm borosilicate glass slide to measure the mechanical beating of cardiomyocytes. The fabrication process of the IDE device has four steps. The first step was cleaning the glass slide: the glass slide was washed with acetone, isopropanol, and deionized water consecutively and dehydrated on a 200 °C hotplate for 10 min. The second step was printing the electrode pattern on the glass slide. Microposit photoresist (S1813, Rohm and Haas) was spin-coated on a glass slide at a speed of 3000 rpm, prebaked for 60 s at 115 °C, exposed to a 20 mW/cm^2^ I-line for 2.5 s, and developed in a Microposit developer (MF CD-26, Shipley) for 40 s. Interdigitated electrode branches were patterned with a 90-µm-diameter circle-on-line and 120 μm center-to-center space of the adjacent branches. The third step was fabricating the conductive layer for the IDE device. A 10-nm-thick Ti layer was sputtered onto the surface of the pattern slide by a thermal evaporator (TE-3, Sharon), and then a 100-nm-thick Au layer was coated. The Ti layer can increase the adhesion between the Au layer and glass slide. The fourth step was removing the conductive layer from the electrode pattern. The glass slide was immersed in acetone and then rinsed with isopropanol and deionized water to remove the photoresist, and the 10 nm Ti layer and 100 nm Au layer were placed on top of the photoresist. An image of the interdigitated electrode branches of our IDE device is shown in Fig. 2b.

### Cardiomyocyte culture

The IDE device was sterilized in 70% ethanol (443611, Sigma–Aldrich) and exposed to UV irradiation for 2 h in a biosafety cabinet. The sterilized IDE device was incubated with 10 mg/ml fibronectin (10838039001, Sigma–Aldrich) in Ca^2+^/Mg^2+^-free phosphate-buffered solution (PBS, P3619, Sigma–Aldrich) at 37 °C with 5.0% CO_2_ for 2 h to improve the biocompatibility of the device surface for cell adhesion. Before cell culture, human-iPSC-CMs (iCell cardiomyocytes 11713, Cellular Dynamic International) were cryopreserved in a vial with liquid nitrogen before cell culture. To thaw the cardiomyocytes, subpackaged cryovials containing 1.5 × 10^6^ cardiomyocytes were immersed and shaken in a 37 °C water bath. The thawed cardiomyocytes were immediately diluted with 10 mL of prechilled medium (iCell Cardiomyocytes Media Kit, Cellular Dynamic International) and moved to a 15 mL centrifuge tube. The medium was centrifuged at 1000 rpm to collect the thawed cardiomyocytes. The collected cardiomyocytes were resuspended in 1 mL of plating medium and seeded in wells of the IDE device at a concentration of 5 × 10^4^ cells per well. After seeding, the IDE device was fixed on the recording system and maintained in an incubator at 37 °C and 5% CO_2_. The culture medium was refreshed every 48 h.

### Detection principle of cardiomyocyte mechanical beating

An IDE-based impedance measurement was applied to detect the mechanical beating of cardiomyocytes. The detection mechanism was based on the variations in the ion current between cardiomyocytes and electrodes during cell attachment, spreading, and proliferation when culturing the cardiomyocytes on the IDEs. Therefore, the mechanical beating of cardiomyocytes can modulate the ion current between cardiomyocytes and electrodes, including weak impedance fluctuations of IDEs, due to changes in cell morphology, cell–cell attachment, and cell-substrate attachment. In the impedance measurement, a low-amplitude sinusoidal signal was conventionally applied on the IDE with a fixed working frequency to generate an ion current between IDE pairs. Feedback of the sinusoidal signal going through the IDEs was detected to calculate the impedance between IDEs. The measured impedance can rhythmically change with the mechanical beating of cardiomyocytes due to rhythmic changes in the ion current. Therefore, the measured IDE impedance variation can represent the mechanical beating of cardiomyocytes.

### Impedance measurement of cardiomyocyte mechanical beating

The multichannel cellular impedance measurement and MNDA together achieved a biosensing system for drug-induced arrhythmia recognition. The biosensing system consisted of a hardware part about data acquisition (DAQ) and a software part about data analysis (Fig. 2c). For the data acquisition, the digital-to-analog converter (DAC) module on the DAQ device generated a sinusoidal signal with an amplitude of 30 mV and a frequency of 10 kHz, which was the input for the IDE device. To match the electrical property of the IDE device, the sinusoidal signal was processed through a signal attenuator and voltage buffer modules before being input into the IDE device. The output sinusoidal current of the IDE device was converted and amplified into an output voltage signal by a transimpedance amplifier. Then, the voltage signal was preprocessed by a high-pass filter, and the impedance was adjusted with a voltage follower and collected by an analog-to-digital (ADC) module on a DAQ device. Digital signal processing was employed to produce mechanical beating signals of cardiomyocytes from the input and output voltage signals with a customized MATLAB program and LABVIEW program. The signal was further denoised by 5-point smoothing filtering to reduce the noise interference for MNDA analysis regarding drug-induced arrhythmia recognition.

### MNDA analysis of cardiomyocyte mechanical beating

Nonlinear dynamics is a great tool to analyze time-domain signals. In this work, we studied whether MNDA recognized arrhythmia from the recording of cardiomyocyte mechanical beating by calculating ten nonlinear dynamic indices, including delay time, correlation dimension, embedding dimension, Kolmogorov entropy, LLE, CO complexity, comentropy, approximate entropy, spectral entropy, and box dimension. The calculation of the MNDA analysis was performed by customized MATLAB programs. The details of the formula of MNDA parameters and the procedures of calculations are described in the supplemental materials.

### Drug assay

Drug assays were conducted after obtaining stable recordings of cardiomyocyte mechanical beating. In this study, the hERG K^+^ channel inhibitor sertindole (HY-14543, MedchemExpress) at a concentration of 2.0 μM was used to treat cardiomyocytes to induce irregular pulsation, while the cardiac stimulant norepinephrine (T7044, Topscience) at concentrations of 80 nM and 400 nM was used to treat cardiomyocytes to induce tachycardia. Before being added to the culture medium, these drugs were dissolved in dimethylsulfoxide (DMSO) and PBS mixed solution and prewarmed in a 37 °C water bath for 5 min.

### Data analysis

Signal processing and principal component analysis (PCA) were realized with a customized LabVIEW and MATLAB program. All statistical analyses were performed using GraphPad Prism 8.0 and OriginPro 2016. All statistical results and error bars are presented as the mean ± standard deviation (SD). Data were analyzed with an unpaired Student’s *t* test, and differences between groups were considered statistically significant when *P* < 0.05.

## Results and discussion

### MNDA parameter calculation and evaluation

**Fig. 3:**
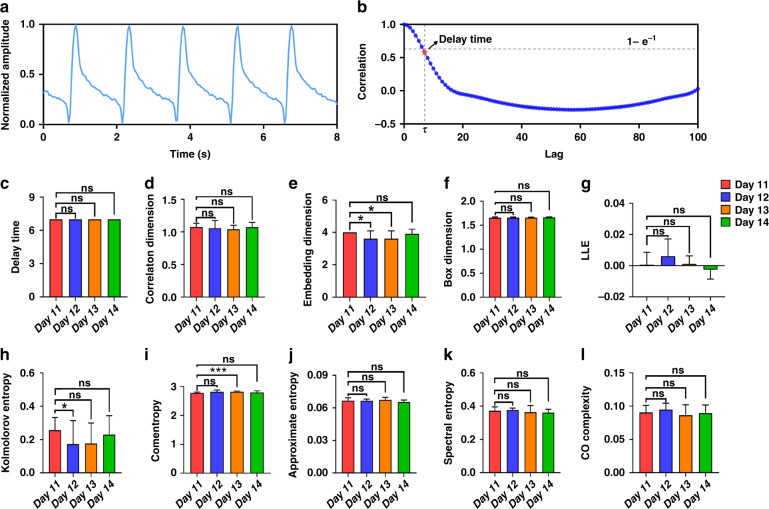
MNDA parameter calculation and evaluation. **a** Cardiomyocyte mechanical beating recording before drug assay. **b** Delay time calculated by the correlation method. The optimal delay time is defined when the autocorrelation method decreases to $1 - \frac{1}{e}$ times its original value. **c**–**l** Statistical comparisons of the MNDA parameters calculated from the beating recordings of cardiomyocyte cultures from the 11th to 14th days. The MNDA parameters consist of **c** delay time, **d** correlation dimension, **e** embedding dimension, **f** box dimension, **g** largest Lyapunov exponent, **h** Kolmogorov entropy, **i** comentropy, **j** approximate entropy, **k** spectral entropy, and **l** CO complexity. Error bars are S.D. and significant differences were performed by *t* test, sample number *n* = 10 recordings for each group, *p < 0.05, **p < 0.01, ***p < 0.001, n.s. not significant

Our work cultured iCell cardiomyocytes on the IDE device and used an impedance measurement system to detect mechanical beating signals of cardiomyocytes. At the beginning of the cardiomyocyte culture, the amplitude and pulse frequency for arrhythmia recognition of the mechanical beating recording was not stable because the impedance measurement was influenced by the proliferation and growth of the cardiomyocyte. After 10 days of culture, the cardiomyocytes stopped proliferating and started beating rhythmically, which enabled mechanical beating recording to show stable amplitude and pulse frequency in most recording channels (Fig. 3a). The recording of cardiomyocyte beating continued to maintain a similar amplitude and pulse frequency over the following days in normal culture without drug treatment (Fig. S1). Thus, the stable mechanical beating recording of the cardiomyocyte culture from the 11th to 14th day was used to explore whether MNDA analysis can provide several parameters to represent the mechanical beating property of cardiomyocytes for arrhythmia recognition in the following work.

The expected MNDA parameters for arrhythmia recognition should be stable from the 11th to 14th day, as we assumed that the parameters were efficiently related to the beating property of cardiomyocytes and were minimally affected by imperceptible and random environmental noise during the recording. Ten commonly used MNDA parameters, including delay time, correlation dimension, embedding dimension, box dimension, LLE, Kolmogorov entropy, comentropy, approximate entropy, spectral entropy, and CO complexity, were extracted from the recording of cardiomyocyte beating and shown in statistics over days (Fig. 3c–i). The statistics reported mean values with standard deviations that suggested stability of MNDA parameters within one day and over multiple days. The ratio between the standard deviation and the mean value of the parameters on the same day revealed the stability of parameters in different measurements within one day. For the stability within one day, both LLE and Kolmogorov entropy showed large fluctuations, with the maximum standard deviation being ~14 times the mean value on the same day (0.00058 ± 0.00798 for LLE on the 11th day). The other eight parameters all showed good stability in different measurements on the same day, and the measurement deviations were always smaller than 10% of the mean value. On the other hand, the mean value change of the MNDA parameters over days suggested the stability of parameters over days. For the stability over days, LLE showed poor stability again and varied more than 10 times in the comparison between parameters extracted from recordings on different days, although no significant difference was observed between different day recordings. The other nine parameters showed good stabilities and varied <10% in the comparison of different day recordings, while the Kolmogorov entropy showed slight variances in different day recordings. Overall, LLE and Kolmogorov entropy were not the appropriate indicators to represent the mechanical beating property of cardiomyocytes as a consequence of their poor stability within one day and over days. The poor performances of LLE and Kolmogorov entropy might be caused by the short time periods (20 s) used in this work since long-term data with several hours or days duration were required by these two parameters. The other eight parameters with good stability can be further screened to find sensitive MNDA parameters for arrhythmia recognition with the recoding of cardiomyocyte beating.

### Sertindole-induced arrhythmia recognition by the biosensing system using MNDA analysis

**Fig. 4:**
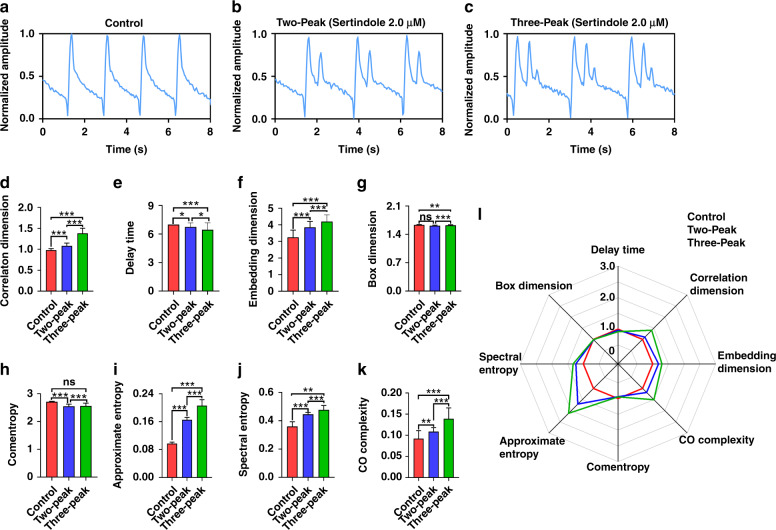
Sertindole-induced arrhythmia recognition by the biosensing system using MNDA analysis. **a**–**c** Cardiomyocyte mechanical beating recording of **a** the control group, **b** the two-peak arrhythmia group, and **c** the three-peak arrhythmia group. The two-peak and three-peak arrhythmias were both induced by the 0.2 μM sertindole treatment. **d**–**k** Statistical comparisons of the MNDA parameters among the control group, the two-peak arrhythmia group, and the three-peak arrhythmia group. The MNDA parameters include **d** delay time, **e** correlation dimension, **f** embedding dimension, **g** box dimension, **h** comentropy, **i** approximate entropy, **j** spectral entropy, and **k** CO complexity. Error bars are S.D. and significant differences were performed by *t*-test, *n* = 10 recordings for each group, *p < 0.05, **p < 0.01, ***p < 0.001, n.s. not significant. **l** The radar map of delay time, correlation dimension, embedding dimension, box dimension, comentropy, approximate entropy, spectral entropy, and CO complexity for the control group, the two-peak arrhythmia group, and the three-peak arrhythmia group. The approximate entropy was the most sensitive to the occurrence of arrhythmia

To explore the capacity of MNDA parameters to recognize arrhythmia from cardiomyocyte beating recordings, we employed an hERG K^+^ channel inhibitor named sertindole to induce arrhythmia and recorded normal cardiomyocyte beating and arrhythmia beating through impedance measurements. Before sertindole treatment, the recordings of cardiomyocyte beating showed normal profiles with stable amplitude and frequency in the control group (Fig. 4a). After 2 μM sertindole treatment, the recording of cardiomyocyte beating showed two or three abnormal peak profiles in one beating and had a longer duration of a single beating from ~2 s to ~3 s (Fig. 4b, c). The recording of cardiomyocyte beating with sertindole treatment provided an arrhythmia sample with multiple distorted peak profiles for MNDA analysis.

To study the sensitivity of the MNDA parameters for sertindole-induced arrhythmia recognition, we selected eight MNDA parameters, including delay time, correlation dimension, embedding dimension, box dimension, comentropy, approximate entropy, spectral entropy, and CO complexity, and extracted these parameters from the cardiomyocyte beating recordings of the control group, two-peak arrhythmia group and three-peak arrhythmia group (Fig. 4d–k). Based on their sensitivity, we classified the MNDA parameters into three categories. The first category included box dimension and comentropy, which showed no significant differences between the control group and one of the multipeak arrhythmia groups. They were not sensitive to the occurrence of arrhythmia and could not serve as indicators for arrhythmia recognition. The second category included delay time, which showed slight differences between the control group and the multipeak arrhythmia groups. There was no obvious increase when the arrhythmia changed from two peaks to three peaks. This implied that the delay time had low sensitivity to the occurrence of arrhythmia with multiple distorted peak profiles and could not recognize the severity of the arrhythmia, considering that the arrhythmia of the three-peak group was more serious than that of the two-peak group. The third category included correlation dimension, embedding dimension, approximate entropy, spectral entropy, and CO complexity, which showed significant differences between the control group and the multipeak arrhythmia groups and revealed more than 10% increments when the arrhythmia changed from two peaks to three peaks. This demonstrated that the five parameters were highly sensitive to the occurrence of arrhythmia with multiple distorted peak profiles and were also sensitive enough to recognize the severity of the arrhythmia. Among the five parameters, the approximate entropy showed the largest increment when the arrhythmia changed from the two-peak group to the three-peak group, making it the best candidate to indicate the severity of the arrhythmia (Fig. 4i). A radar map collected the responses of eight MNDA parameters together and showed that the approximate entropy was the most sensitive parameter to indicate the occurrence of the arrhythmia (Fig. 4l). Therefore, approximate entropy enabled the MNDA analysis to recognize the arrhythmia with distorted peaks and evaluate the severity of the arrhythmia through cardiomyocyte beating recording.

### NE-induced arrhythmia recognition using MNDA analysis

**Fig. 5:**
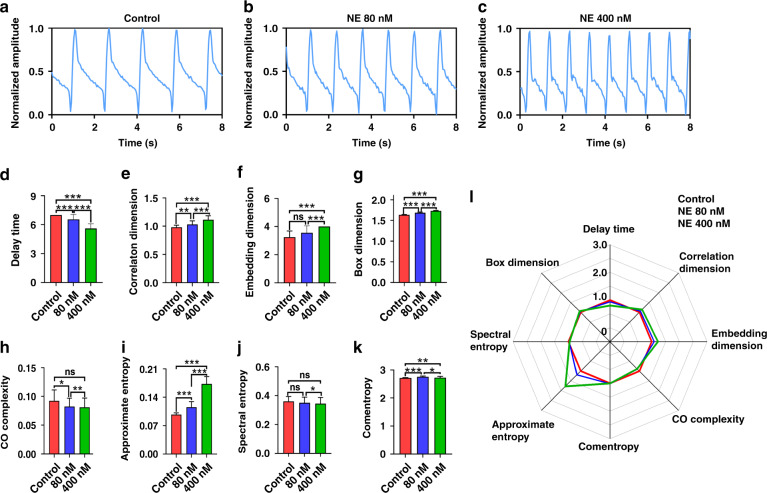
NE-induced arrhythmia recognition by the biosensing system with MNDA analysis. **a**–**c** Cardiomyocyte mechanical beating recording of **a** the control group, **b** the 80 nM NE-treated arrhythmia group, and **c** the 400 nM NE-treated arrhythmia group. The NE-treated arrhythmia groups both showed a faster beating rate than the control, but they did not show distorted peaks in the beating. **d**–**k** Statistical comparisons of the MNDA parameters among the control group, the 80 nM NE-treated arrhythmia group, and the 400 nM NE-treated arrhythmia group. The MNDA parameters include **d** delay time, **e** correlation dimension, **f** embedding dimension, **g** box dimension, **h** comentropy, **i** approximate entropy, **j** spectral entropy, and **k** CO complexity. Error bars are S.D. and significant differences were performed by *t* test, *n* = 10 recordings for each group, *p < 0.05, **p < 0.01, ***p < 0.001, n.s. not significant. **l** The radar map of delay time, correlation dimension, embedding dimension, box dimension, comentropy, approximate entropy, spectral entropy, and CO complexity for the control group, the two-peak arrhythmia group, and the three-peak arrhythmia group. The approximate entropy was the most sensitive to the occurrence of the arrhythmia

In addition to the arrhythmia with distorted multipeak profiles, some arrhythmias showed abnormal beating rates in the mechanical beating recording of cardiomyocytes after drug treatment. To further test the performance of MNDA analysis in recognizing these arrhythmias with abnormal beating rates, the current study employed a hormone signaling substance named NE at concentrations of 80 nM and 400 nM to induce the arrhythmia. Before NE treatment, the recording of cardiomyocyte beating showed a normal beating rate of ~0.6 s^−1^, which was similar to that of the control group (Fig. 5a). After 80 nM NE treatment, the recording of cardiomyocyte beating showed a quicker beating rate of ~0.8 s^−1^, which was 30% faster than that of the control group (Fig. 5b). Increasing the NE concentration of the treatment made the beating rate much faster in the cardiomyocyte beating recording. The beating rate reached ~1.1 s^−1^ with NE treatment at 400 nM. The beating rate of the 400 nM NE-treated group was more than 100% faster than that of the control group (Fig. 5c). Both the 80 nM and 400 nM NE-treated groups did not show significant multipeak distortions in the individual beating of the recording. The recording of cardiomyocyte beating with NE treatment provided an arrhythmia sample whose beating rate was faster than normal and whose peak was not distorted on profiles for MNDA analysis.

To study the sensitivity of the MNDA parameters for NE-induced arrhythmia recognition, we obtained eight MNDA parameters from the cardiomyocyte beating recordings of the control group, 80 nM NE-treated group, and 400 nM NE-treated group (Fig. 5d–k). Based on their sensitivities, the MNDA parameters can also be classified into three categories. The first category consisted of embedding dimension, CO complexity, and spectral entropy, which showed no significant differences between the control group and one of the NE-treated groups. These three MNDA parameters of the first category were nonsensitive to the occurrence of the arrhythmia and cannot be taken as the indicators for NE-treated arrhythmia recognition. The second category contained box dimension and comentropy, which showed significant differences between the control group and two NE-treated groups, although there was little difference between the 80 nM NE-treated group and 400 nM NE-treated group. It is implied that these two parameters of the second category had low sensitivity to beating rate change of arrhythmia and did not have sufficient sensitivity to recognize the arrhythmia included by NE at different concentrations. The third category included delay time, correlation dimension, and approximate entropy, which showed significant differences between the control group and the two NE-treated groups. These parameters were significantly different between the 80 nM NE-treated group and the 400 nM NE-treated group. The approximate entropy showed the largest increment when the NE concentration of the treatment changed from 80 nM to 400 nM and the beating rate of the recording changed from 0.8 s^−1^ to 1.1 s^−1^. This result suggested that the approximate entropy maintained a strong positive correlation with the beating rate that indicated the severity of the arrhythmia (Fig. 5i). A radar map collected responses of the eight MNDA parameters to the NE-induced arrhythmia together and showed that the approximate entropy was the most sensitive parameter to describe the occurrence and severity of the arrhythmia. This was the same as the statistical results. Thus, the approximate entropy allowed the MNDA analysis to recognize the arrhythmia with an abnormal quick beating rate from the mechanical beating recording of cardiomyocytes.

### Arrhythmia classification by the biosensing system using MNDA analysis

**Fig. 6:**
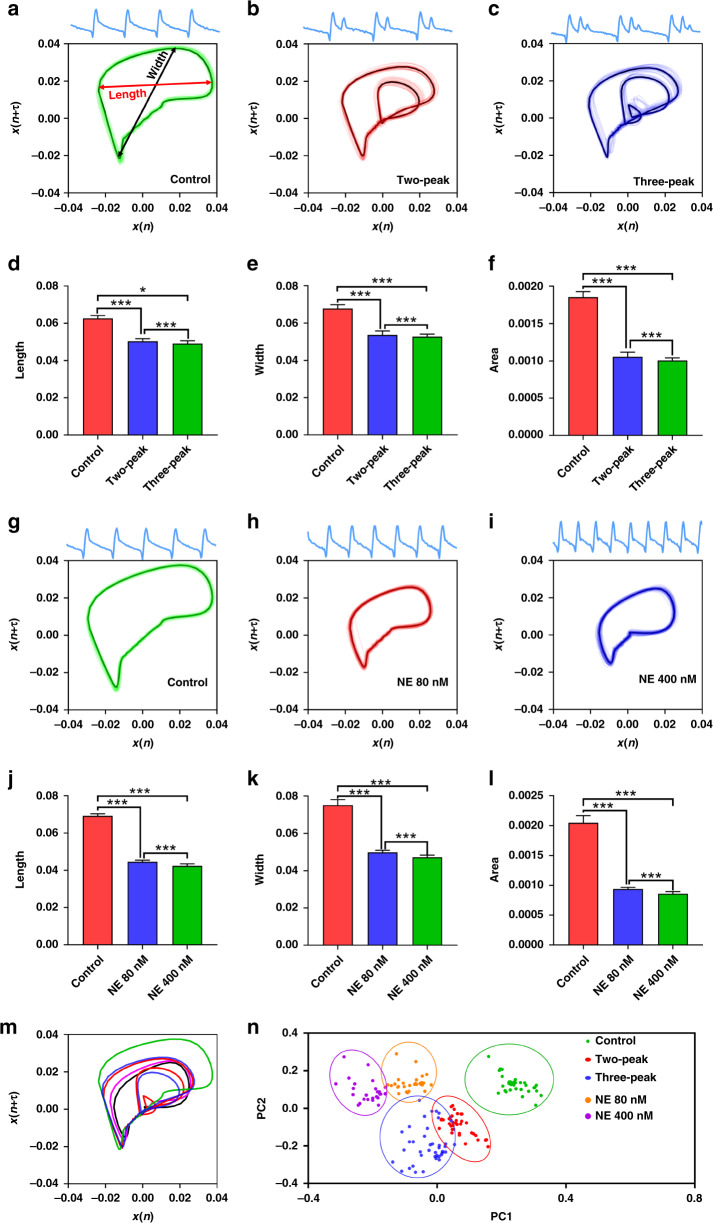
Arrhythmia classification by the biosensing system with MNDA analysis. **a**–**c** Two-dimensional phase space reconstruction of **a** the control group, **b** the two-peak arrhythmia group, and **c** the three-peak arrhythmia group. The two-peak and three-peak arrhythmias were both induced by the 0.2 μM sertindole treatment. **d**–**f** Statistical comparisons of the shape features of the reconstructed plotting among the control group, the two-peak arrhythmia group, and the three-peak arrhythmia group. The shape features include **d** length, **e** width, and **f** area of the reconstructed plot. **g**–**i** Two-dimensional phase space reconstruction of **g** the control group, **h** the 80 nM NE-treated arrhythmia group, and **i** the 400 nM NE-treated arrhythmia group. **j**–**l** Statistical comparisons of the shape features of the reconstructed plot among the control group, the 80 nM NE-treated arrhythmia group, and the 400 nM NE-treated arrhythmia group. The shape features include **j** length, **k** width, and **l** area of the reconstructed plot. **n** Two-dimensional phase space reconstructions of all types of cardiomyocyte beating recordings, including the control group (green), the two-peak arrhythmia group (red), the three-peak arrhythmia group (blue), the 80 nM NE-treated arrhythmia group (orange), and the 400 nM NE-treated arrhythmia group (purple), in one graph. The two-peak and three-peak arrhythmias were both induced by the 0.2 μM sertindole treatment. **m** The PCA clustering of the MNDA reconstructed plots of the control group, the two-peak arrhythmia group, the three-peak arrhythmia group, the 80 nM NE-treated arrhythmia group, and the 400 nM NE-treated arrhythmia group. Error bars are S.D. and significant differences were performed by *t* test, *n* = 10 recordings for each group, *p < 0.05, **p < 0.01, ***p < 0.001, n.s. not significant

After demonstrating the validity of the approximate entropy of MNDA analysis in recognizing the occurrence of arrhythmia, we further explored whether MNDA analysis can distinguish different arrhythmias and classified the different arrhythmias into several groups. Two-dimensional phase space reconstruction was applied to analyze the arrhythmia induced by various drugs at different concentrations. The reconstruction converted cardiomyocyte beating signals in the time domain into phase space graphs in the two-dimensional space domain by setting *x(n)* serial as the x-coordinate and *x(n* + *τ)* serial as the y-coordinate when *τ* was the delay time from autocorrelating calculation (Fig. 6a, see Methods). For sertindole-induced arrhythmia with distorted peak profiles, the phase space graphs showed different plotting for the control group, the two-peak group, and the three-peak group. There were two and three circles in the graph of the two-peak group and three-peak group, respectively; however, there was only a single circle in the graph of the control group (Fig. 6a–c). The number and size of the circles in the graph were strongly associated with the number and the size of the peak in one beating, suggesting that the phase space reconstruction has well-represented arrhythmia information in the two-dimensional space plotting. The imaging process can further extract length, width, and area information from the graphs to compare the control group and arrhythmia groups. There were significant differences between the control group and the arrhythmia groups in all three statistics in length, width, and area information, although there was little difference between the two arrhythmia groups. The results suggested that the two-dimensional phase space reconstruction allowed MNDA analysis to graphically recognize the occurrence of the sertindole-induced arrhythmia with distorted peaks, while the reconstruction graph could not quantify and classify different degrees of the arrhythmias.

We also used two-dimensional phase space reconstruction to analyze NE-included arrhythmias that had undistorted peaks and faster beating rates than normal samples (Fig. 6g–i). There was only one circle in all phase space graphs of the control group, 80 nM NE-treated group, and 400 nM NE-treated group because the recording of all groups showed only one peak in one beating. The results were consistent with those of the sertindole-induced arrhythmia analysis. The graphical analysis showed that the area of the circles in the graph was strongly related to the beating rate of the arrhythmia. Hence, we further extracted length, width, and area information from the graphs to perform comparisons between the control group and the NE-induced arrhythmia groups. The comparison indicated that there were significant differences between the control group and the arrhythmia group in all three statistics regarding the length, width, and area information (Fig. 6j–l). However, the differences between the two NE-induced arrhythmias were not easy to distinguish, similar to the graphical analysis of the sertindole-induced arrhythmia. The results suggested that the MNDA analysis using phase space reconstruction recognized the occurrence of NE-induced arrhythmia with a fast-beating rate and partly classified the severity of the arrhythmia.

To classify different arrhythmias well, we plotted all the two-dimensional phase space reconstructed curves of the control groups, the sertindole-induced arrhythmia group, and the NE-induced arrhythmia group in one graph (Fig. 6m). In the graph, the curves of the arrhythmia groups always showed smaller areas than those of the control group and showed high diversity in shape details across group members. To quantify the difference, we employed principal component analysis (PCA) to reduce the dimensionality of the curves and minimize the information loss of the curves for the arrhythmia classification. The PCA created two new uncorrelated variables, PC1 and PC2, which can successfully classify the control group and four arrhythmia groups into five major clusters (Fig. 6n). Distances between the clusters of the control group and the four arrhythmia groups showed a strong linear relationship with the severity of the arrhythmias (Figs. 6n and S2). For sertindole-induced arrhythmia, the distance between the control group and the three-peak group was ~2.5 times the distance between the control group and the three-peak group. For NE-induced arrhythmia, the distance between the control group and 400 nM NE-treated group was approximately twice the distance between the control group and 80 nM NE-treated group. All the results obviously suggested that the PCA for the two-dimensional phase space plotting of MNDA analysis can recognize different arrhythmias and semiquantify the severity of the arrhythmias.

## Discussion

Our work showed a biosensing system that combined MNDA analysis and the IDE technique to recognize drug-induced arrhythmias from the cardiomyocyte beating signals. This is the first report to apply nonlinear dynamics analysis to identify drug-induced arrhythmias based on cardiomyocyte mechanical beating signals. The IDE impedance measurement provided a multichannel recording of the mechanical beating of cardiomyocytes. After testing ten commonly used MNDA parameters, our work screened out an arrhythmia-sensitive MNDA parameter, approximate entropy, which can efficiently analyze the recording and reliably indicate the occurrence of drug-induced arrhythmia. In the test, the two drugs, sertindole, and NE, induced two types of arrhythmias that possessed distorted peak profiles and abnormally fast-beating rates, respectively. We found that the correlation dimension and approximate entropy were the only two MNDA parameters that were sensitive to both the distorted peak profiles and the abnormally fast-beating rates of the arrhythmias. Among the two parameters, the approximate entropy showed a large increment with the severity of the arrhythmias, providing a quantitative parameter to indicate the severity of the arrhythmias. Compared to the previously reported automated template matching^28^, the quantification using the approximate entropy avoided the comparison between the recording and the control template through the one-by-one matching of the beating waveform. This usage of the MNDA parameter to analyze the recording of cardiomyocyte beating further simplified the steps of recognizing drug-induced arrhythmias and improved the efficiency of recognition over large datasets. This biosensing system also identified the difference between 80 nM norepinephrine and the control with significance *p* < 0.001 using the parameter approximate entropy. This result demonstrated the sensitivity of our biosensing system to low drug concentrations.

Our work demonstrated the application of MNDA analysis in distinguishing different arrhythmias with several drug treatments. The MNDA parameter, approximate entropy, can recognize drug-induced arrhythmias but fails to distinguish arrhythmias induced by different drug treatments. This limitation on arrhythmia distinguishing also existed in a previously reported method using automated template matching to recognize arrhythmias^28,40^. To overcome this limitation, our work employed two-dimensional phase space reconstruction in MNDA analysis for drug-induced arrhythmia classification based on cardiomyocyte beating recording. The reconstruction converted the long time-series recording into the graphical plotting in the limited spatial space. The graphical plotting provided significant differences to distinguish the arrhythmias induced by different drugs or by the same drugs at different concentrations. Combined with PCA, the plotting classified the arrhythmias into several major clusters in a two-dimensional space and thus successfully distinguished the arrhythmias that were induced by different drugs. Furthermore, the arrhythmia classification clearly grouped the clusters of the arrhythmias induced by the same drug. The distances between the same drug-induced arrhythmia clusters and the control cluster increased with the severity of the arrhythmia and therefore can quantify the severity of the arrhythmia, which is crucial to drug development. Another limitation of our work is that only two drugs, sertindole, and norepinephrine, were studied. However, the arrhythmia-like traces induced by these drugs covered most of the representative traces, including the two-peak trace, multipeak trace, and accelerated trace presented by Blinova et al.^41^, which used 28 drugs with a range of proarrhythmic capacities. To improve the capacity of the biosensing system in identifying different drug-induced arrhythmias, artificial intelligence-based methods will be combined with the MNDA. More drugs will be introduced to test the capacity of this improved biosensing system. Overall, the application of MNDA in analyzing cardiomyocyte beating recordings provides a promising way to distinguish different arrhythmias and quantify the severity of arrhythmias.

Apart from cardiomyocyte beating recording, our work can also influence the analysis of other electrophysical and electrophysiological recordings. The MNDA can potentially be applied in other biosensing systems to recognize cardiac diseases^42–45^. Of the MNDA parameters, approximate entropy can precisely recognize the occurrence of drug-induced arrhythmia, and the MNDA phase space reconstruction can reliably distinguish drug-induced arrhythmias. The MNDA reconstruction can also quantify the severity of drug-induced arrhythmias, which is useful for drug development. MNDA analysis can also benefit the signal processing of other physiological recordings (e.g., calcium transients of neurons and action potentials of cardiomyocytes) that have been led by the one-by-one matching of individual waveforms in screen distorted profiles^46–48^. Therefore, the application of MNDA analysis in signal processing is likely to improve the data analysis efficiency and thereby boost our understanding of the recording. This MNDA-based biosensing system also showed advantages compared to other cell-based methods that analyze arrhythmias (Table S1). Our work provides a new and promising approach to analyze physiological recordings, especially cardiac‐related signals.

## Conclusion

In summary, we reported a biosensing system utilizing MNDA analysis to recognize and classify drug-induced arrhythmias from cardiomyocyte beating signals recorded by IDE impedance measurements. This biosensing system can successfully recognize the data features of arrhythmias via the screened MNDA parameter of approximate entropy, distinguish different arrhythmias by two-dimensional phase space reconstruction in MNDA analysis, and quantify the severity of arrhythmias by combining MNDA reconstruction and PCA clustering. This new biosensing system is a promising tool for recognizing and classifying drug-induced arrhythmias in cardiological and pharmaceutical applications.

## Supplementary information

SUPPLEMENTAL MATERIAL_A biosensing microsystem using multiparameter nonlinear dynamic analysis of cardiomyocyte beating for drug-induced arrhythmia recognition
