# Supplementary material for: A biosensing system using a multiparameter nonlinear dynamic analysis of cardiomyocyte beating for drug-induced arrhythmia recognition
Source: Microsyst Nanoeng. 2022 May 9;8:49. doi: 10.1038/s41378-022-00383-1 (PMC9081091; doi:10.1038/s41378-022-00383-1)
Supplement: Supplementary file 1 — SUPPLEMENTAL MATERIAL_A biosensing microsystem using multiparameter nonlinear dynamic analysis of cardiomyocyte beating for drug-induced arrhythmia recognition [file 41378_2022_383_MOESM1_ESM.docx]

**Supplementary Information**

**A biosensing system using multiparameter nonlinear dynamic analysis of cardiomyocyte beating for drug-induced arrhythmia recognition**

Hao Wang ^a^^†^, Yue Wu ^b†^, Quchao Zou ^c†^, Wenjian Yang ^b^, Zhongyuan Xu ^b^, Hao Dong ^b^, Zhijing Zhu ^d, e^, Depeng Wang ^f^, Tianxing Wang ^g^, Ning Hu ^a, c, h*^, Diming Zhang ^b*^

^a^State Key Laboratory of Optoelectronic Materials and Technologies, Guangdong Province Key Laboratory of Display Material and Technology, School of Electronics and Information Technology, Sun Yat-sen University, Guangzhou 510006, China.

^b^Research Center for Intelligent Sensing Systems, Zhejiang Lab, Hangzhou, 311121, China

^c^ZJU-Hangzhou Global Scientific and Technological Innovation Center, Department of Chemistry, The Second Affiliated Hospital Zhejiang University School of Medicine, Department of Clinical Medical Engineering, Zhejiang University, Hangzhou, 310058, China.

^d^Key Laboratory of Novel Target and Drug Study for Neural Repair of Zhejiang Province, School of Medicine, School of Computer & Computing Science, Zhejiang University City College, Hangzhou, 310015, China

^e^School of Brain Science and Brain Medicine, Zhejiang University, Hangzhou, 310058, China

^f^College of Energy and Power Engineering, Nanjing University of Aeronautics and Astronautics, Nanjing, 210016, China

^g^E-LinkCare Meditech Co., Ltd., Hangzhou, 310011, China

^h^State Key Laboratory of Transducer Technology, Chinese Academy of Sciences, Shanghai, 200050, China.

*Corresponding author: Diming Zhang ([zhangdm@zhejianglab.edu.cn](mailto:zhangdm@zhejianglab.edu.cn)) or Ning Hu ([huning@zju.edu.cn](mailto:huning@zju.edu.cn) or [huning3@mail.sysu.edu.cn](mailto:huning3@mail.sysu.edu.cn))

^†^These co-first authors contributed equally to this work


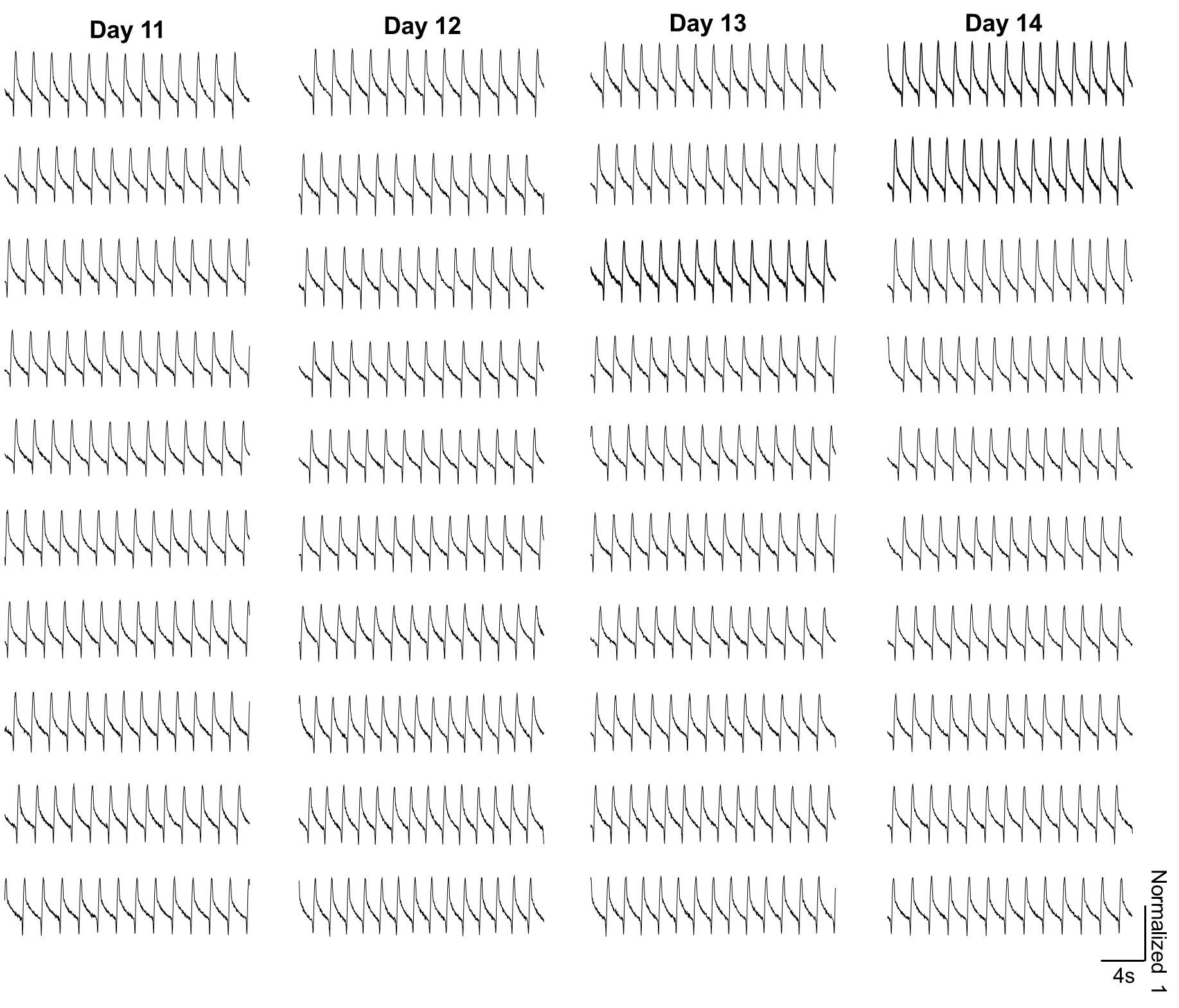


**Figure S1 -** Recording of cardiomyocyte beating from 11^th^ to 14^th^ days of the cardiomyocyte culture. All traces were normalized to the first spike of each trace.


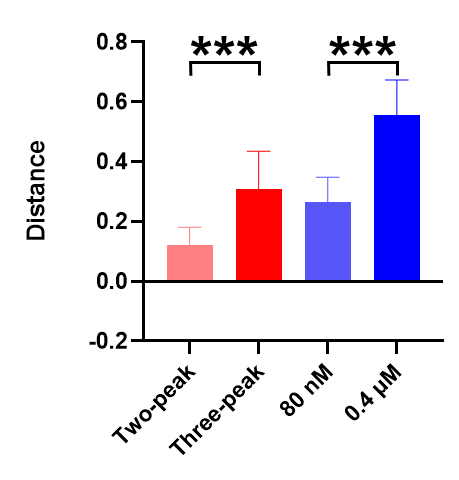


**Figure S2.** Distances between the clusters of the control group and four arrhythmia groups in the PCA classification. *** denotes p<0.001.

**Table S1. The common cell-based methods to analyze the arrhythmias.**

| Method | Label or invasion | Throughput | Long-term  recording | Accuracy | Real-time | Ref. |
| --- | --- | --- | --- | --- | --- | --- |
| Multi-well patch clamp | Yes | Low/High | No (Tens of minutes) | High | Yes | ^2,3^ |
| Traditional MEAs | No | High | Yes (Tens of days) | Low | Yes | ^4,5^ |
| Nanostructured MEAs | Yes | High | No (Tens of Hours) | High | Yes | ^4,5^ |
| Video-based analysis without dye | No | High | Yes (Tens of days) | Low | No | ^6,7^ |
| Video-based analysis with dyes | Yes | High | No (Tens of days) | High | No | ^8,9^ |
| MNDA combined with IDE | No | High | Yes (Tens of days) | High | Yes | This work |

**Calculation of MNDA analysis**

In the calculation of MNDA analysis, the recording of cardiomyocyte beating works as a one-dimensional discrete digital series in time domain and defined as $\left\{ x\left( i \right), i=0, 1 ,2 , \ldots, N \right\}$ when *N* is the length of the digital series. The calculation of delay time, correlation dimension, embedding dimension, Kolmogorov entropy, largest Lyapunov exponent, CO complexity, comentropy, approximate entropy, spectral entropy and box dimension are listed below.

1. **Phase space reconstruction**

The phase space is a space in which all possible states of the system are represented, with each possible state corresponding to one unique point. The phase space is therefore reconstructed to extract some valuable features by extending a one-dimensional time series to a high dimensional phase space. For a time series $\left\{ x\left( i \right),i=1,2, \ldots, N \right\}$**, the phase space points** can be reconstructed as ^1^:

|  | $\left\{ X\left( i \right)\boldsymbol{,}1\leq i\leq n-\left( m-1 \right) \right\}\boldsymbol{,}and X\left( i \right)=x\left( i \right),x\left( i+\tau\right)\ldots,x\left( i+\left( m-1 \right)\tau\right)$ | $\left( Eq.1 \right)$ |
| --- | --- | --- |

**where** $\tau$ **is the delay time,** $N$ **is the length of the time series and** $m$ **is the embedding dimension. If we set the embedding dimension** $m$ **as 2, the time series reconstructed in the phase space can be written as follows:**

|  | $\left\{ X\left( i \right)\boldsymbol{,}1\leq i\leq n-1 \right\}\boldsymbol{,}and X\left( i \right)=x\left( i \right), x\left( i+\tau\right)$ | $\left( Eq.2 \right)$ |
| --- | --- | --- |

Therefore, $x\left( i \right)$ **and** $x\left( i+\tau\right)$ **can be used as the X axis and Y axis to plot the two-dimensional phase space graph.**

1. **Delay time**

The delay time $\tau$ was calculated by autocorrelation method which determined the optimal time delay according to the autocorrelation function. The autocorrelation function was denoted as the following equation:

$$\begin{aligned} R_{xx}\left( u \right)=\frac{1}{N}\sum_{i=0}^{N-1} x\left( i \right)x\left( i+u \right),\#\left( \mathrm{Eq}.3 \right) \end{aligned}$$

where $R_{xx}\left( u \right)$ is the autocorrelation coefficient of the digital series$\left\{ x\left( i \right), i=0, 1 ,2 , \ldots, N \right\}$. The optimal delay time was defined as the value when the autocorrelation coefficient is $1-\frac{1}{e}$ times of its original value $R_{xx}\left( 0 \right)$. The optimal delay point number *τ* was calculated as follows:

$$\begin{aligned} \tau=\arg min(R_{xx}\left( u \right)-(1-\frac{1}{e})R_{xx}\left( 0 \right)) ,\#\left( \mathrm{Eq}.4 \right) \end{aligned}$$

1. **Correlation dimension and embedding dimension**

In this work, the correlation dimension was calculated by Grassberger-Procaccia (G-P) algorithm ^2^. Time series $\left\{ x\left( i \right), i=1 ,2 , \ldots,N \right\}$ was reconstructed in phase space as:

$$\begin{aligned} \left\{ X\left( i \right)\boldsymbol{,}1\leq i\leq N-\left( m-1 \right) \right\}\boldsymbol{,}and X\left( i \right)=\left[ x\left( i \right),x\left( i+\tau\right)\ldots,x\left( i+\left( m-1 \right)\tau\right) \right],\boldsymbol{\#}\left( \mathrm{Eq}.5 \right) \end{aligned}$$

**where** $\tau$ **is the delay time,** $N$ **is the length of the time series and** $m$ **is the embedding dimension. The length of**$X\left( i \right)$ **was defined** as $N_{m}=N-\left( m-1 \right)$**.** **We calculated correlation integral** $C_{m}^{2}(r)$ **by the following equation:**

$$\begin{aligned} C_{m}^{2}\left( r \right)= \frac{1}{N_{m}\left( N_{m}-1 \right)} \sum_{i=1}^{N_{m}} \sum_{\begin{aligned} j=1 \\ j\neq i \end{aligned}}^{N_{m}} H\left( r-r_{ij} \right),\#\left( \mathrm{Eq}.6 \right) \end{aligned}$$

**where** $r$ **is the phase space scale,** $r_{ij}$ **is the Chebyshev distance between the arbitrarily two point of** $\left\{ X\left( i \right),1\leq i\leq n-\left( m-1 \right) \right\}$ **and follows the equation:**

$$\begin{aligned} r_{ij}=||X\left( i \right)-X\left( j \right) ||={[\sum_{l=0}^{m-1} \left( x\left( i+l\tau\right)-x\left( j+l\tau\right) \right)^{2}]}^{\frac{1}{2}}.\#\left( \mathrm{Eq}.7 \right) \end{aligned}$$

$H$ **is the Heaviside function as shown below:**

$$\begin{aligned} H\left( x \right)= \left\{ \begin{aligned} 1 x\geq0 \\ 0 x<0 \end{aligned} \right..\#\left( Eq.8 \right) \end{aligned}$$

**In a certain range, the correlation integral** $C(r)$ **had a subscript relation with *r* which was described in the following equation:**

$$\lim_{r\to0} C\left( r \right)\propto r^{d} (Eq.9)$$

**Thus, the correlation dimension *d* was calculated as follows:**

$$\begin{aligned} d=\lim_{r\to0} \frac{\ln C\left( r \right)}{\ln r}.\#\left( Eq.10 \right) \end{aligned}$$

**In this study, the initial value of embedding dimension** $m$ **was set as 2 and incrementally increased until the value of the correlation dimension did not change. The value of correlation dimension was the final results of our correlation dimension calculation by the G-P algorithm. According to the embedding dimension theory, the relation between correlation dimension and embedding dimension should satisfy the equation below:**

$$\begin{aligned} m>2d+1,\#\left( Eq.11 \right) \end{aligned}$$

**In this study, the embedding dimension** $m$ **was selected from the minimum integers that satisfied** $m>2d+1$ **when the correlation dimension *d* followed the maximum principle above.**

1. **Kolmogorov entropy**

**The input of the** Kolmogorov entropy **algorithm was a time series** $\left\{ x\left( i \right),i=1, 2, \ldots, N \right\}$**.** In this work, the Kolmogorov entropy was also calculated by the G-P algorithm ^3^. The time series$\left\{ x\left( i \right),i=1, 2, \ldots, N \right\}$ was reconstructed in phase space by Eq.4 and we repeated the calculation from Eq.5 to Eq.7.

In the G-P algorithm, the second order Rényi entropy $K_{2}$ was used as an approximation of Kolmogorov entropy. The relation between $K_{2}$ **entropy and correlation integral** $C_{m}^{2}\left( r \right)$ **was shown as follows:**

$$\begin{aligned} K_{2}= \lim_{r\to0} \lim_{N_{m}\to\infty}\frac{1}{m\tau}\log_{2} \frac{C_{m}\left( r \right)}{C_{m+1}\left( r \right)}.\#\left( \mathrm{Eq}.12 \right) \end{aligned}$$

**The Kolmogorov entropy calculation was divided into three steps. First, we obtained the selecting the phase space scale** $r$ **from the standard deviation** $\sigma$ **of the time series. The phase space scale** $r$ **satisfied the following equation in the non-scaling region of figure** $\ln C_{m}\left( r \right) \sim\ln\left( r \right)$**:**

$$\frac{\sigma}{2}\leq r\leq\sigma\left( \mathrm{Eq}.13 \right)$$

**Second, we set he phase space scale *r* as its maximum** $\sigma$ **and increased the** **embedding dimension** $m$ **at 2 with step of 1 until the** $K_{2}$ **entropy did not change with the increase of *m*. The value of embedding dimension was recorded as *m_1_*.**

**Third, we decreased the phase space scale** $r$ **in the non-scaling region of figure** $\ln C_{m}\left( r \right) \sim\ln\left( r \right)$**, when the embedding dimension was set as *m_1_*. The value of the** $K_{2}$ **entropy was determined when the entropy** $K_{2}$ **did not change with the decrease of the phase space scale** $r$**. We used the** $K_{2}$ **as the result of the** **Kolmogorov entropy calculation in this study.**

1. **Largest Lyapunov exponent**

**The input of the** **largest Lyapunov exponent algorithm was a time series** $\left\{ x\left( i \right),i=1,2, \ldots, N \right\}$**. According to the phase space reconstruction theory, the time series** $\left\{ x\left( i \right), i=0, 1 ,2 , \ldots, N \right\}$ **could also be reconstructed by** Eq.4.

**Largest Lyapunov Exponent was calculated by** **Rosenstein algorithm ^4^. First, we set the initial point** $X(i)$ **of time** $t_{i}$**, and found its nearest electricity. After time of** $k\Delta t$**, the distance between these two points became** $d_{k}(i)$**. We repeated the first step for the whole time series to calculate the average distance as the following:**

$$\begin{aligned} \bar{d}_{k}=\frac{1}{M}\sum_{i-1}^{i=M} d_{k}\left( i \right).\#\left( Eq.14 \right) \end{aligned}$$

**We repeated the above steps to obtain the average distance** $\bar{d}_{k}$ **corresponding to different time interval** $k\Delta t$**. We drew a** $\ln\bar{d}_{k}$ **vs.** $k\Delta t$ **curve and used the least square method to fit the curve. The slope of the curve is the maximum Lyapunov exponent.**

1. **CO complexity**

**The input of the** **CO complexity algorithm was a time series** $\left\{ x\left( i \right),i=1,2, \ldots, N \right\}$**. The calculations were as follows ^5^. First, we calculated Fourier transform of the time series as follows:**

$$\begin{aligned} \hat{X}\left( j \right)=\sum_{k=0}^{N-1} x\left( k \right)e^{-\frac{2\pi ikj}{N}}, j=0 ,1 ,2 ,\ldots, N-1,\#\left( Eq.15 \right) \end{aligned}$$

**where** $i=\sqrt{-1}$ **is an imaginary unit. The mean square value of** $\left\{ \hat{X}\left( j \right), j=0, 1 ,2 , \ldots, N-1 \right\}$ **can be calculated as follows:**

$$\begin{aligned} G_{N}= \frac{1}{N}\sum_{j=0}^{N-1} \left| \hat{X}\left( j \right) \right|^{2},\#\left( Eq.16 \right) \end{aligned}$$

**Second, we defined** $\tilde{X}\left( j \right)$ **by equation Eq.16 and calculated Fourier inversion transform of** $\left\{ \tilde{X}\left( j \right), j=0, 1 ,2 , \ldots, N-1 \right\}$ **by equation Eq.17:**

$$\begin{aligned} \tilde{X}\left( j \right)= \left\{ \begin{aligned} \hat{X}\left( j \right)，when \left| \hat{X}\left( j \right) \right|^{2}\leq G_{N} \\ 0，when \left| \hat{X}\left( j \right) \right|^{2}> G_{N} \end{aligned} \right.,\#\left( Eq.17 \right) \end{aligned}$$

$$\begin{aligned} \tilde{X}\left( k \right)=\frac{1}{N}\sum_{j=0}^{N-1} \hat{X}\left( j \right)e^{\frac{2\pi ikj}{N}}, k=0 ,1 ,2 ,\ldots, N-1,\#\left( Eq.18 \right) \end{aligned}$$

**Third, we calculated the CO complexity by the following equation:**

$$\begin{aligned} C_{o}= \frac{\sum_{k=0}^{N-1} \left| \tilde{X}\left( k \right) \right|^{2}}{\sum_{k=0}^{N-1} \left| x\left( k \right) \right|^{2}}.\#\left( \mathrm{Eq}.19 \right) \end{aligned}$$

1. **Comentropy**

**The input of the** **Comentropy entropy algorithm was a time series** $\left\{ x\left( i \right),i=1,2, \ldots, N \right\}$**. The Comentropy was calculated by the following formula ^6^:**

$$\begin{aligned} H\left( X \right)=-\sum_{x\in X} p\left( x \right)\log p\left( x \right),\#\left( Eq.20 \right) \end{aligned}$$

**where** $p\left( x \right)$ **is the probability that** $x$ **appears in the time series** $\{x\left( i \right),i=0,1,2, \ldots, N\}$**.**

1. **Approximate entropy**

**The input of the** **approximate entropy algorithm was a time series** $\left\{ x\left( i \right),i=1,2, \ldots, N \right\}$**. The calculations consisted of four steps showed as follows ^7^. First, we split the time series**$x\left( i \right)$ **with the time window** $m$ **into** $N-m+1$ **subseries as denoted by the equation Eq.4. First, we calculated the distances between individual sequences and all other** $N-m+1$ **sequences, and built a table in which each element was described by the following equation:**

$$\begin{aligned} d_{ij}=max\left| x_{i}\left( t \right)- x_{j}\left( t \right) \right|, \#\left( Eq.21 \right) \end{aligned}$$

**wh**ere $i$ and $j$ are both equal to$1 ,2 , \ldots, N-m+1,$ $t$ is equal to $1, 2, \ldots, i+m-1$**, and** *d_ij_* represents **the maximum value of the absolute values of the difference subseries.**

**Second, we defined a threshold *r* and calculated statistic ratio of the numbers that were smaller than *r* in each row of the table by the following equation:**

$$C_{i}^{m}\left( r \right)=\frac{num[d_{ij}<r]}{N-m+1}. (Eq.22)$$

**Third, we got the logarithmic average of** $C_{i}^{m}\left( r \right)$ **as follows**

$$\begin{aligned} \emptyset^{m}\left( r \right)=\frac{1}{n-m+1}\sum_{i=1}^{n-m+1} \ln C_{i}^{m}\left( r \right),\#\left( Eq.23 \right) \end{aligned}$$

**Fourth, we increased window length** $m$ **to** $m$**+1 and repeated the above steps from Eq.18 to Eq. 20 to get** $\emptyset^{m+1}\left( t \right)$**. The approximate entropy was calculated as the difference between** $\emptyset^{m}\left( t \right)$ **and** $\emptyset^{m+1}\left( t \right)$**:**

$$\begin{aligned} ApEn\left( t \right)= \emptyset^{m}\left( r \right)-\emptyset^{m+1}\left( r \right),\#\left( Eq.24 \right) \end{aligned}$$

**In this study, we set *m* as 2 and *r* as 0.2**×***SD*. *SD* is standard deviation of the time series.**

1. **Spectral entropy**

**The input of the spectral entropy algorithm was a time series** $\left\{ x\left( n \right),n=1,2, \ldots, N \right\}$ **with length** $N$**. The calculations included five steps showed as follows ^8^. First, we removed the direct current component of** $x\left( n \right)$ **as follows:**

$$\begin{aligned} x\left( n \right)= x\left( n \right)-\bar{x},\#\left( Eq.25 \right) \end{aligned}$$

**where** $\bar{x}$ **is the mean of** $x\left( n \right)$**. Second, we calculated the Fourier transform of** $x\left( n \right)$ **as follows:**

$$\begin{aligned} X\left( k \right)=\sum_{n=0}^{N-1} x\left( n \right)e^{-\frac{2\pi nkj}{N}}, \#\left( Eq.26 \right) \end{aligned}$$

**where** $k=1,2,\ldots, N-1$**and** $j=\sqrt{-1}$ **is an imaginary unit. Third, we calculated the power spectrum value of a certain frequency point** $p\left( k \right)$ **by the following equation:**

$$\begin{aligned} p\left( k \right)=\frac{1}{n}\left| X\left( k \right) \right|^{2},where k=1,2,\ldots,\frac{N}{2},\#\left( Eq.27 \right) \end{aligned}$$

**At the same time, we calculated the total power** $P_{tot}$ **of the** $X\left( k \right)$ **sequence as follows:**

$$\begin{aligned} P_{tot}=\frac{1}{N}\sum_{k=1}^{\frac{N}{2}} \left| X\left( k \right) \right|^{2},\#\left( Eq.28 \right) \end{aligned}$$

**Fourth, the relative power spectrum probability of the sequence** $P_{k}$ **was calculated by the following equation:**

$$\begin{aligned} P_{k}=\frac{p\left( k \right)}{P_{tot}}=\frac{\frac{1}{N}\left| X\left( k \right) \right|^{2}}{\frac{1}{N}\sum_{k=1}^{\frac{N}{2}} \left| X\left( k \right) \right|^{2}},\#\left( Eq.29 \right) \end{aligned}$$

**Fifth, the Spectral entropy**$se$ **was calculated by:**

$$\begin{aligned} se= - \sum_{k=1}^{\frac{N}{2}} P_{k}\ln P_{k}.\#\left( Eq.30 \right) \end{aligned}$$

1. **Box dimension**

**The input of the box dimension algorithm was a time series** $\left\{ x\left( i \right),i=1,2, \ldots, N \right\}$ **with length** $N$**. The calculations included three steps showed as follows ^9^. First, we resampled the signal time series with temporal interval *T* into the frequency domain signal with frequency interval** ${1/\sigma}_{t}$**. The relationship between the temporal interval *T* and frequency interval** ${1/\sigma}_{t}$ **followed the equation:**

$${T/\sigma}_{t}=2^{k}+1, (Eq. 31)$$

**where** $k=1,2, \ldots, N$**. Then, we scaled the amplitude in proportion to make the amplitude of the frequency domain between 0 and** $2^{k}$**. The signal sample point sequence after processing became** $\left\{ S\left( n \right),n=1,2, \ldots,2^{k}+1 \right\}$**.**

**Second, we set** $\sigma_{i}$ **in grid scale variation as follows:**

$\sigma_{i}=2^{i-1}\sigma_{t}, \left( i = 1,2,\ldots,M; M\leq k \right), (Eq. 32)$

$\sigma_{1}$**is equal to** $\sigma_{t}$**, and the signal sample sequence is divided into** $L=2^{k+1-i}$ **segments when covering** $S$ **with a square grid of side length** $\sigma_{i}$**. The minimum number of grids covering** $S$ **was denoted as** $N(\sigma_{i})$**:**

$$N\left( \sigma_{i} \right)=\frac{1}{2^{i-1}}\sum_{j=1}^{L} |\max\left\{ S\left( \left( j-1 \right)*2^{i-1}+1 \right),\ldots,S\left( j*2^{i-1}+1 \right) \right\}-\min\left\{ S\left( \left( j-1 \right)*2^{i-1}+1 \right),\ldots,S\left( j*2^{i-1}+1 \right) \right\}|, \left( Eq. 33 \right)$$

**Third, we set** $x_{i}=\log\left( 2^{k+1-i} \right)$ **and** $y_{i}=\log N\left( \sigma_{i} \right)$**, and plotted out** $M$ **point pairs**${(x}_{i}, y_{i})$**. The point pairs** ${(x}_{i}, y_{i})$ **were fitted to be a straight line by the least square method. The slope of the line is the estimation of box dimension** $D_{B}$**:**

$$\begin{aligned} D_{B}=\frac{\left( \sum_{i=1}^{M} y_{i} \right)\left( \sum_{i=1}^{M} x_{i} \right)-M\sum_{i=1}^{M} {y_{i}x}_{i}}{\left( \sum_{i=1}^{M} x_{i} \right)^{2}-M\sum_{i=1}^{M} {x_{i}}^{2}},\#\left( Eq. 34 \right) \end{aligned}$$

**The** $x_{i}$ **and** $y_{i}$ **was further substituted into the estimated value of binary box dimension. Then, the box dimension** $D_{B}$ **was calculated as follows:**

$$\begin{aligned} D_{B}=\frac{M\sum_{i=1}^{M} \log N\left( \sigma_{i} \right)\log2^{i}-\sum_{i=1}^{M} \log N\left( \sigma_{i} \right)\sum_{i=1}^{M} \log2^{i}}{\left( \sum_{i=1}^{M} \log2^{i} \right)^{2}-M\sum_{i=1}^{M} \left( \log2^{i} \right)^{2}}.\#\left( Eq. 35 \right) \end{aligned}$$

**Reference**

1 Henry, B., Lovell, N. & Camacho, F. Nonlinear dynamics time series analysis. *Nonlinear biomedical signal processing: Dynamic analysis and modeling* **2**, 1-39, doi:10.1109/9780470545379.ch1 (2001).

2 Cutler, C. D., Grenfell, B. T., May, R. M. & Tong, H. A theory of correlation dimension for stationary time series. *Philosophical Transactions of the Royal Society of London. Series A: Physical and Engineering Sciences* **348**, 343-355, doi:doi:10.1098/rsta.1994.0095 (1994).

3 Grassberger, P. & Procaccia, I. Estimation of the Kolmogorov entropy from a chaotic signal. *Physical review A* **28**, 2591, doi:10.1103/PhysRevA.28.2591 (1983).

4 Rosenstein, M. T., Collins, J. J. & De Luca, C. J. A practical method for calculating largest Lyapunov exponents from small data sets. *Physica D: Nonlinear Phenomena* **65**, 117-134, doi:<https://doi.org/10.1016/0167-2789(93)90009-P> (1993).

5 En-hua, S., Zhi-jie, C. & Fan-ji, G. Mathematical foundation of a new complexity measure. *Applied Mathematics and Mechanics* **26**, 1188-1196, doi:10.1007/BF02507729 (2005).

6 Shannon, C. E. A mathematical theory of communication. *The Bell system technical journal* **27**, 379-423, doi:10.1002/j.1538-7305.1948.tb01338.x (1948).

7 Pincus, S. M. Approximate entropy as a measure of system complexity. *Proceedings of the National Academy of Sciences* **88**, 2297-2301, doi:10.1073/pnas.88.6.2297 (1991).

8 Zhang, A., Yang, B. & Huang, L. in *2008 international conference on BioMedical engineering and informatics.* 435-439 (IEEE).

9 Elezović, N., Županović, V. & Žubrinić, D. Box dimension of trajectories of some discrete dynamical systems. *Chaos, Solitons & Fractals* **34**, 244-252, doi:<https://doi.org/10.1016/j.chaos.2006.03.060> (2007).
